# Infective Costochondritis after Augmentation Mammoplasty: A Rare Case Report and Review of the Literature

**DOI:** 10.1055/a-2088-2829

**Published:** 2023-09-08

**Authors:** Sally Min, Jinil Choi, Kwon Joong Na, Ki Yong Hong

**Affiliations:** 1 Department of Plastic and Reconstructive Surgery, Seoul National University College of Medicine, Seoul National University Hospital, Seoul, Republic of Korea; 2 Department of Thoracic and Cardiovascular Surgery, Seoul National University Hospital, Seoul, Republic of Korea

**Keywords:** infective costochondritis, augmentation mammoplasty, surgical debridement, chest wall reconstruction, case report

## Abstract

Silicone breast implant insertion is a commonly performed surgical procedure for breast augmentation or reconstruction. Among various postoperative complications, infection is one of the main causes of patient readmission and may ultimately require explantation. We report a case of infective costochondritis after augmentation mammoplasty, which has rarely been reported and is therefore difficult to diagnose. A 36-year-old female visited the clinic for persistent redness, pain, and purulent discharge around the left anteromedial chest, even after breast implant explantation. Magnetic resonance imaging showed abscess formation encircling the left fourth rib and intracartilaginous and bone marrow signal alteration at the left body of the sternum and left fourth rib. En bloc resection of partial rib and adjacent sternum were done and biopsy results confirmed infective costochondritis. Ten months postoperatively, the patient underwent chest wall reconstruction with an artificial bone graft and acellular dermal matrix. As shown in this case, early and aggressive surgical debridement of the infected costal cartilage and sternum should be performed for infective costochondritis. Furthermore, delayed chest wall reconstruction could significantly contribute to the quality of life.

## Introduction

Silicone breast implant insertion is a commonly used surgical procedure for breast augmentation and reconstruction after mastectomy. Among the various postoperative complications after breast implantation, infection is one of the main causes of patient readmission, which may require explantation of the implant. The incidence of postoperative infection after breast implantation is reported to be approximately 2.5 to 6%, 1 2 3 which presents as redness, swelling, pain, tenderness, or discharge. If an implant infection is clinically suspected, treatment usually starts with immediate empirical antibiotic therapy, and surgical intervention such as debridement or pocket irrigation can be performed. 3 Unfortunately, when the infection persists or relapses even after management, implant explantation is the ultimate treatment choice.

Although prosthetic infection is a well-known complication of breast implantation, infective costochondritis is rare and is therefore poorly recognized. To the best of our knowledge, few cases of infective costochondritis were previously reported in the literature. 4 5 Here, we present a rare case of infective costochondritis after augmentation mammoplasty and describe the treatment course.

## Case

**Fig. 1:**
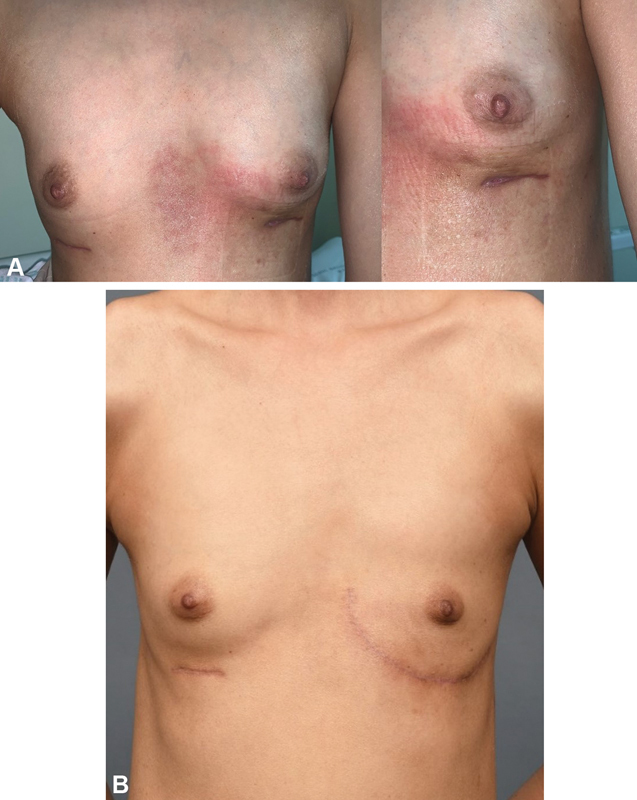
Initial medical photo ( **A** ) and medical photo taken 7 months after initial surgical debridement ( **B** ).

**Fig. 2:**
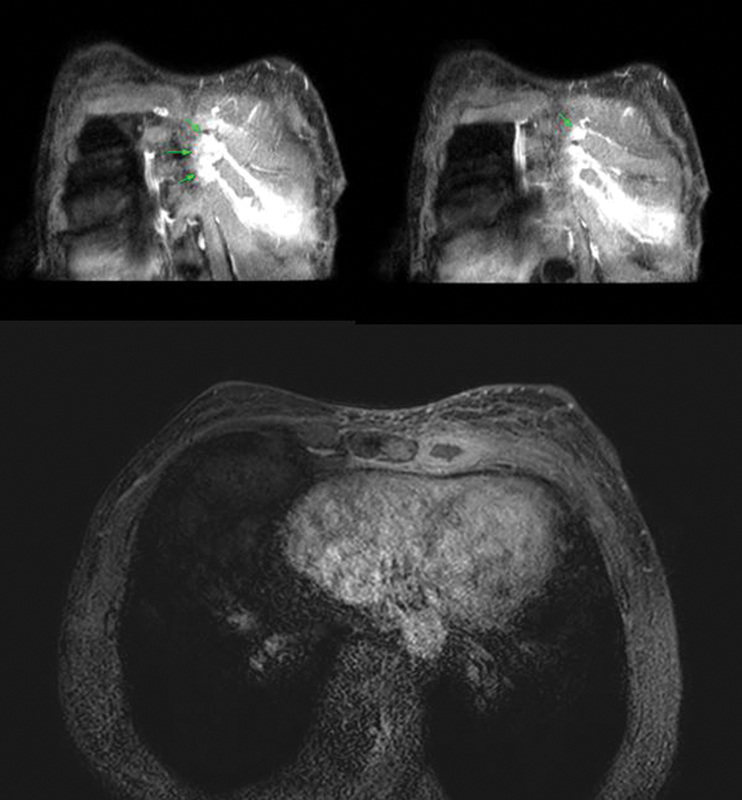
Initial breast magnetic resonance imaging at coronal and axial view has shown increased extent of bone marrow enhancement at left sternal body to fourth costosternal junction and perichondrial enhancement around left costal cartilage, probably due to infection.

A 36-year-old female visited the clinic with persistent redness, pain, and tenderness around the left anteromedial chest combined with purulent discharge from a previous surgical wound ( Fig. 1 ). Initially, the patient underwent bilateral subpectoral augmentation mammoplasty via an inframammary fold incision. However, left-sided chest pain started 1 week after augmentation and persisted for 2 weeks. Oral antibiotics was given for 2 weeks; however, the symptoms and signs did not improve; hence, bilateral breast implant removal was performed. During surgery, seropurulent periprosthetic fluid was found after which meticulous pocket irrigation and debridement of infectious, necrotic tissue were done. There was no sign of secondary infection of bony structures. However, unlike common implant infections, symptoms aggravated even after explantation and she sustained antibiotic use for 3 months. The patient specifically complained of pain around midsternum area where redness, swelling, and yellowish purulent discharge were accompanied. Her C-reactive protein levels were mildly elevated at 2.35 mg/dL (normal range, 0–0.9 mg/dL) and no leukocytosis was shown (6.78 × 10 ^9^ /L). She showed no fever, maintaining her body temperature below 37.0°C during admission. Magnetic resonance imaging (MRI) revealed 5.6 × 1.5 × 7.2 cm extent of infiltrative rim-enhancing mass formation encircling the left fourth rib, involving the left parasternal area to left breast lower area, suggesting abscess formation ( Fig. 2 ). Intracartilaginous and bone marrow signal alteration at the left body of sternum and left fourth rib was also shown, which implicated the possibility of osteomyelitis or costochondritis. The cooperation was instantly decided by the thoracic surgeons.

**Fig. 3:**
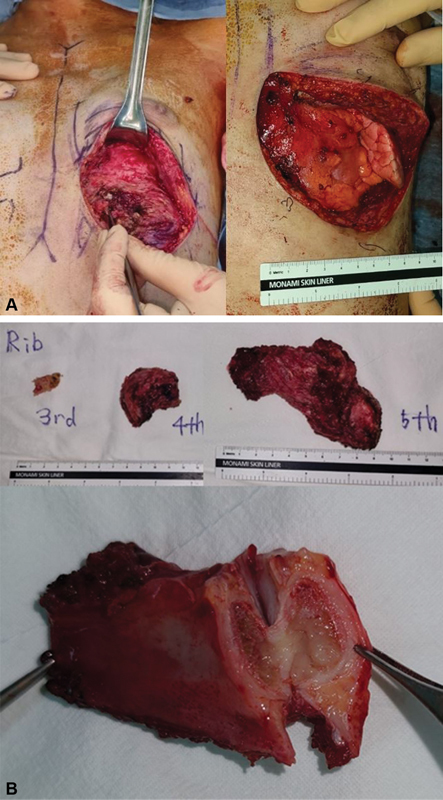
Partial resection of infectious fourth and fifth rib bone, cartilage, and adjacent partial sternum were done, after which lung was partially exposed ( **A** ). Resected specimen of rib and sternum were sent for microbiological examination ( **B** ).

Intraoperatively, an infectious tract was found along the fourth intercostal space to the lower margin of the left fourth rib. We performed total fistulectomy, and thoracic surgeons performed en bloc resection of the partial left fourth and fifth rib and partial sternum, ranging from the cartilage portion to the anterior axillary line ( Fig. 3A ). Although postoperative flail chest was a concern, chest wall reconstruction using a prosthesis was not performed at the time due to the high infection risk. Permanent biopsy results confirmed costochondritis and intraoperative tissue culture was reported negative ( Fig. 3B ). She was maintained on 6 weeks of oral amoxicillin clavulanate postoperatively.

**Fig. 4:**
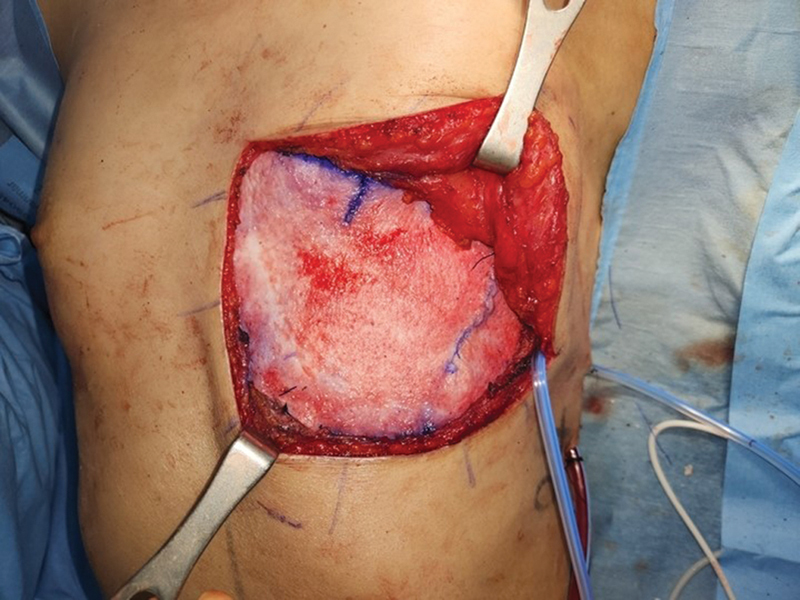
After chest wall reconstruction with bone cement and mesh, acellular dermal matrix (BellaCell HD, 8cm  × 16 cm) was inserted for fascial layer coverage.

After 10 months, when the wound was stabilized, she underwent chest wall reconstruction with bone cement and nonabsorbable mesh (Bard Soft Mesh Nonabsorbable Polypropylene Monofilament) using the sandwich technique. Cooperation was performed by plastic surgeons, in which an ADM onlay graft (BellaCell HD, 8 × 16 cm) was followed for fascia closure ( Fig. 4 ). After prosthesis insertion, there was no recurrence of infection until postoperative 3 months, and computed tomography of the chest at postoperative 1 month presented no signs of infection.

## Discussion

Costochondritis after breast augmentation is a rare complication; hence, its pathogenesis is not yet established. It is important to differentiate between inflammatory and infective costochondritis since each has its different treatment strategy. Inflammatory costochondritis is self-limiting and responds well to anti-inflammatory drugs and steroids. It is also often associated with a particular connective tissue disease, which is the Tietze syndrome. However, infective costochondritis invariably requires surgical management, and therefore should be diagnosed early enough to prevent further infection of deeper structures such as the pleura or lung. It could be difficult to differentiate infective costochondritis from usual infectious prosthetic complication. Although implant infection usually subsides after explantation, the patient consistently complained of focal pain and discharge around midsternum area even after implant removal in this case. C-reactive protein levels were mildly elevated at 2.35 mg/dL (normal range, 0–0.9 mg/dL) without leukocytosis, and she showed no fever. These atypical manifestations necessitated additional imaging study for diagnosis, which in this case we proceeded MRI scan. The following case highlights the importance of early diagnosis and intervention of infective costochondritis. Surgeons should undergo immediate radical debridement of the infected rib cartilage and partial sternum to prevent recurrence.

Several theories have been suggested by previous studies regarding the pathogenesis of infectious costochondritis. Interestingly, most of all infectious cases reported in the literature, including the present case, occurred following subpectoral implant placement. 4 6 7 8 9 This is probably due to the fact that intraoperative exposure of subpectoral structures is necessary for bony contamination. It is difficult to identify the specific event that causes infection; however, we hypothesized that the primary infection of the periprosthetic compartment subsequently caused a secondary infection of the sternocostal joint and rib in a delayed period, despite initial appropriate management had been done for usual postoperative prosthetic infection. Since the clinical course and aggressiveness of costochondritis relates to virulence of the involved microflora, microbiological findings could help explain the infection route.

In previous cases of rib chondritis or osteomyelitis after breast implant insertion, the collected bone specimens were found to be positive for *Staphylococcus aureus* , *Staphylococcus epidermidis, or Pseudomonas aeruginosa* . 4 5 6 7 8 9 In fact, these microorganisms are known as the most common pathogens of early infection after breast implant insertion, which implies the possibility of secondary infection caused by prosthesis contamination. We used amoxicillin–clavulanic acid as empiric antibiotic in this case, which is reported active against pathogens commonly found in prosthesis-related bone infections, especially targeting methicillin-susceptible *S. aureus* and *S. epidermidis* . 10 Although we sustained its use since no organism was cultured from our bone specimen, further antibiotic regimen change could be made according to culture results. Also, the fact that no organism was cultured from the intraoperative bone specimen is noteworthy among previous case reports. It is presumably due to the effect of prolonged antibiotic use on culture sensitivity. This also implies the importance of early surgical intervention with collection of intraoperative tissue culture, since prolonged use of antibiotics could only influence the accuracy of future culture results.

Considering that previous studies have mostly focused on the primary surgical treatment of infective costochondritis, the present case is notable for its additional reconstruction of bone and chest wall defects. After discharge, the patient persistently complained of severe discomfort due to flail chest along with prominently sunken anterior chest at inhalation and directly palpable heart. Although chest wall reconstruction using two-layer mesh and bone cement sandwich technique is frequently performed in thoracic surgery after oncologic resection or trauma, there is few literature reporting its application for secondary treatment of infective costochondritis. Subsequent reconstruction is not a necessity, but we suggest that it could improve quality of life for patients suffering from postoperative chest wall deformities. In fact, this procedure was a key factor contributing to patient satisfaction in the present case. Moreover, we believe that in this case, a two-stage operation with delayed reconstruction using a prosthesis was an appropriate choice in terms of reducing the risk of infection. In this context, immediate prosthesis insertion should be reconsidered in clinical settings in which the infection has not been completely resolved.

Rib costochondritis after breast augmentation is rare and difficult to diagnose. Early diagnosis of rib costochondritis with imaging studies is important, and instant surgical debridement as needed, followed by delayed chest wall reconstruction should be performed. Further research is needed to determine the exact pathogenesis of bony infections after breast implant insertion.
